# Supplementary material for: Pre-exposure prophylaxis uptake concerns in the Democratic Republic of the Congo: Key population and healthcare workers perspectives
Source: PLoS One. 2023 Nov 2;18(11):e0280977. doi: 10.1371/journal.pone.0280977 (PMC10621847; doi:10.1371/journal.pone.0280977)
Supplement: S1 Table — (DOCX) [file pone.0280977.s001.docx]

S1 Table: Healthcare workers (HCW) self-reported gaps of improving key population pre-exposure prophylaxis (PrEP) service, in the Democratic Republic of the Congo, February to November 2018

The question in the HCW survey: What in your opinion can be done to improve services for key populations in health facilities in DRC?

|  | **HCW PrEP Survey (N=30)** |
| --- | --- |
| Additional training of HCWs, N (%) | 19 (63%) |
| Development of new policies, N (%) | 14 (47%) |
| Revision of laws/regulations on KP, N (%) | 15 (50%) |
| Introducing special clinic hours for KP, N (%) | 11 (37%) |
| Additional sensitization on KP*, N (%) | 17 (57%) |
| Clinical competency in providing care for KP, N (%) | 23 (77%) |
| How to communicate with KP, N (%) | 20 (67%) |

*Focused skills building on addressing the needs of KP clients in healthcare settings
