# Supplementary material for: Pre-exposure prophylaxis uptake concerns in the Democratic Republic of the Congo: Key population and healthcare workers perspectives
Source: PLoS One. 2023 Nov 2;18(11):e0280977. doi: 10.1371/journal.pone.0280977 (PMC10621847; doi:10.1371/journal.pone.0280977)
Supplement: S1 File — (PDF) [file pone.0280977.s002.pdf]

**KP Client Survey Questionnaire**

## SURVEY QUESTIONNAIRE

*INTRODUCTION:* Thank you again for agreeing to participate in our survey. Some of the questions you will be asked are about private things and could make you feel shy or uncomfortable. You can choose not to answer any questions that you do not want to. You may also ask me to explain questions if you do not understand them. If you do not know the exact answer to a question, try to give it your best guess. For most questions, choose the one answer that best fits. We will tell you if a question allows for more than one answer. You may also stop the interview at any time if you decide you no longer want to participate. Please remember that your responses to our questions are identified only by a number not linked to your name or any clinic records, so your responses are completely anonymous. Your name and clinic ID numbers do not appear on this questionnaire.
*Data collector to assess participant eligibility before proceeding to survey questions.*

### Eligibility assessment Section

| ELIGIBILITY QUESTIONS | | | | |
|---|---|---|---|---|
| **No.** | **QUESTIONS & INSTRUCTIONS** | **RESPONSES** | | **SKIPS** |
| **1.** | Do you consider yourself: male, female, transgender or other? | 1. Male<br>2. Female<br>3. Transgender (male to) Female<br>4. Transgender (female to) Male<br>5. Other________________<br>6. Refused | 0<br>1<br>2<br>3<br>4<br>-2 | |
| **2.** | What was your sex at birth? | 1. Male<br>2. Female<br>3. Other ________________<br>4. Refused | 0<br>1<br>2<br>-2 | |

| ELIGIBILITY QUESTIONS | | | | |
|---|---|---|---|---|
| **No.** | **QUESTIONS & INSTRUCTIONS** | **RESPONSES** | | **SKIPS** |
| **3.** | Do you have sex with: | 1. Men only<br>2. Women only<br>3. Both men and women<br>4. Refused | 0<br>1<br>2<br>-1 | |
| **4.** | Is selling sex your main source of income? | 1. Yes<br>2. No<br>3. Don't know<br>4. Refused | 1<br>0<br>-1<br>-2 | |
| **5.** | In the last 6 months, have you injected illicit or illegal drugs? | 1. Yes<br>2. No<br>3. Don't know<br>4. Refused | 1<br>0<br>-1<br>-2 | |
| **6.** | Have you ever taken PrEP (which is a medication to help prevent you from getting HIV)? You may be currently taking it or have in the past. | Yes<br>No<br>Don't know | 1<br>0<br>-2 | *If No or Don't know,* ***STOP*** *survey.*<br>*If Yes, continue with survey* |
| **7.** | Have you completed a survey at this clinic about your experience with PrEP use | Yes<br>No | 1<br>0 | *If Yes,* ***STOP*** *survey.*<br>*If No, continue with survey.* |

*STOP*

*To be classified in SurveyCTO :*

| **Key Population Classification:** | |
|---|---|

| | |
|---|---|
| **If client answers Male to Q1 and answers Men Only or Men and Women to Q3, then classify as MSM** | □ |
| **If client answers Transgender MTF or FTM to Q1, or if client identifies as a gender different from their birth sex, then classify as TG** | □ |
| **If client answers Yes to Q4, then categorize as SW** | □ |
| **If client answers Yes to Q5, then classify as PWID** | □ |
| **Final Classification: (*mark *ALL* that apply*) □MSM □TG □SW □PWID**<br>***Some clients may belong to more than one category due to overlapping vulnerabilities and behavior**<br><br>**If no above boxes were marked (MSM, TG, SW, PWID), STOP Survey** | |

*FOR INTERVIEWER:* Patient must be classified as being part of a KP group **AND** having initiated PrEP (it may include those currently taking PrEP and those who have discontinued) in order to be eligible to continue with the survey.

| No. | QUESTIONS & INSTRUCTIONS | RESPONSES | | SKIPS |
|---|---|---|---|---|
| *Section A: Socio-demographics* | | | | |
| **A01** | How old were you at your last birthday? | [ ][ ] Years<br>Don't know<br>Refused | -1<br>-2 | |
| **A02** | Have you ever attended school? | Yes<br>No<br>Don't know<br>Refused | 1<br>0<br>-1<br>-2 | *If Yes, Go to Q A03*<br>*If No, Go to Q A04* |

| No. | QUESTIONS & INSTRUCTIONS | RESPONSES | | SKIPS |
|---|---|---|---|---|
| **A03** | What is the highest level of formal schooling you completed? | Did not complete elementary school<br>(Grade 1 to Grade 5) | 0 | |
| | | Completed elementary school<br>(Grade 1 to Grade 5) | 1 | |
| | | Did not complete middle school<br>(Grade 6 to Grade 9) | 2 | |
| | | Completed middle school<br>(Grade 6 to Grade 9) | 3 | |
| | | Did not complete high School<br>(Grade 10 to Grade 11) | 4 | |
| | | Completed high school but not graduated<br>from college/university | 5 | |
| | | Graduated college/university | 6 | |
| | | Post-graduate education | 7 | |
| | | Other (Specify: e.g. vocational | 8 | |
| | | training)____________________ | -1 | |
| | | Don't know | -2 | |
| | | Refused | | |
| **A04** | Are you currently working for regular income? | Yes | 1 | |
| | | No | 0 | |
| | | Refused | -2 | |
| **A05** | What is your current marital status? | Currently married or living together | 0 | |
| | | Separated / divorced & not currently living<br>with partner | 1 | |
| | | Widowed & not currently living with | 2 | |
| | | partner | 3 | |
| | | Never married & not currently living with | | |
| | | partner | 4 | |
| | | Other_________________________ | -1 | |
| | | Don't know | -2 | |
| | | Refused | | |

| No. | QUESTIONS & INSTRUCTIONS | RESPONSES | | SKIPS |
|---|---|---|---|---|
| *Section B : PrEP Uptake, Adherence, and Satisfaction* | | | | |
| **No.** | **QUESTIONS & INSTRUCTIONS** | **RESPONSES** | | |
| **B01** | Are you currently taking PrEP? | Yes<br>No<br>Don't know | 1<br>0<br>-1 | *If No, Go to Q B05* |
| **B02** | In the last 7 days, on how many days did you miss taking your PrEP medicine? | [ ][ ]<br>Number of days<br><br>Don't know<br>Refused | <br><br><br>-1<br>-2 | |
| **B03** | In the last 7 days, how good a job did you do at taking your PrEP medicine in the way you were supposed to? | Very poor<br>Poor<br>Fair<br>Good<br>Very good<br>Excellent<br>Don't know<br>Refused | 0<br>1<br>2<br>3<br>4<br>5<br>-1<br>-2 | |
| **B04** | How satisfied are you with your prescribed PrEP regimen? | Satisifed<br>Somewhat satisified<br>Somewhat unsatisified<br>Unsatisifed<br>Don't know<br>Refused | 0<br>1<br>2<br>3<br>-1<br>-2 | |
| **B05** | When did you stop taking PrEP? | [ ][ ] [ ][ ][ ][ ]<br>Month Year<br><br>Don't know<br>Refused | <br><br><br>-1<br>-2 | |

| No. | QUESTIONS & INSTRUCTIONS | RESPONSES | | SKIPS |
|---|---|---|---|---|
| **B06** | Why did you stop taking PrEP? | Tested postive for HIV<br>No longer engaging in “at-risk behaviors”<br>You experienced too many side effects from taking PrEP<br>Your life is so busy you do not have time to take PrEP<br>You have no time to go to the clinic for follow-up PrEP visits<br>Other________________________<br>Don’t know<br>Refused | 1<br>2<br>3<br>4<br>5<br>6<br>7<br>-1<br>-2 | |
| *Section C: Perceived PrEP Benefits and Barriers* | | | | |
| Please tell me your reactions to the following statements.<br><br>*If you take PrEP regularly…* | | | | |
| **C01** | you would suffer from side effects | Strongly Disagree<br>Disagree<br>Agree<br>Strongly Agree<br>Don’t Know<br>Refused | 0<br>1<br>2<br>3<br>-1<br>-2 | |
| **C02** | you will avoid the shame of developing HIV | Strongly Disagree<br>Disagree<br>Agree<br>Strongly Agree<br>Don’t Know<br>Refused | 0<br>1<br>2<br>3<br>-1<br>-2 | |
| **C03** | you won’t develop HIV and won’t give it to other people | Strongly Disagree<br>Disagree<br>Agree<br>Strongly Agree<br>Don’t Know<br>Refused | 0<br>1<br>2<br>3<br>-1<br>-2 | |

| No. | QUESTIONS & INSTRUCTIONS | RESPONSES | | SKIPS |
|---|---|---|---|---|
| **C04** | you would be taking control of your life | Strongly Disagree<br>Disagree<br>Agree<br>Strongly Agree<br>Don't Know<br>Refused | 0<br>1<br>2<br>3<br>-1<br>-2 | |
| **C05** | you would be concerned that others may think you have HIV | Strongly Disagree<br>Disagree<br>Agree<br>Strongly Agree<br>Don't Know<br>Refused | 0<br>1<br>2<br>3<br>-1<br>-2 | |
| **C06** | you would set a good example for others | Strongly Disagree<br>Disagree<br>Agree<br>Strongly Agree<br>Don't Know<br>Refused | 0<br>1<br>2<br>3<br>-1<br>-2 | |
| **C07** | your family and friends will shun you because they may think you have HIV | Strongly Disagree<br>Disagree<br>Agree<br>Strongly Agree<br>Don't Know<br>Refused | 0<br>1<br>2<br>3<br>-1<br>-2 | |
| **C08** | you would stay healthy and not get sick from HIV | Strongly Disagree<br>Disagree<br>Agree<br>Strongly Agree<br>Don't Know<br>Refused | 0<br>1<br>2<br>3<br>-1<br>-2 | |
| **C09** | it may affect your health | Strongly Disagree<br>Disagree<br>Agree<br>Strongly Agree<br>Don't Know<br>Refused | 0<br>1<br>2<br>3<br>-1<br>-2 | |

| No. | QUESTIONS & INSTRUCTIONS | RESPONSES | | SKIPS |
|---|---|---|---|---|
| **C10** | it may decrease the effectiveness of HIV medicines if you do become HIV-positive | Strongly Disagree<br>Disagree<br>Agree<br>Strongly Agree<br>Don't Know<br>Refused | 0<br>1<br>2<br>3<br>-1<br>-2 | |
| **C11** | Many people have a hard time taking medications exactly when and how they are recommended (prescribed). In the past 7 days, please tell if any of these made it HARD OR HARDER for you to take PrEP every day, regardless of whether or not you actually took the pills? Select all that apply. | I ran out of pills<br>I forgot<br>I was worried about others thinking I have HIV because they saw me taking the pill<br>I was worried about experiencing side effects<br>I had side effects<br>I was worried about mixing the study pills with drugs or alcohol<br>I was drunk<br>I was high on drugs<br>I just did not feel like taking it/did not want to be bothered by it<br>I felt the pills were not needed<br>Other, specify: ________________<br>Don't know<br>Refused | 0<br><br>2<br>3<br>4<br><br>5<br>6<br>7<br><br>8<br>9<br>10<br><br>11<br><br>12<br><br>13<br>-1<br>-2 | |
| *Section D: Clinic experiences* | | | | |

| No. | QUESTIONS & INSTRUCTIONS | RESPONSES | | SKIPS |
|---|---|---|---|---|
| **D01** | At your visit for PrEP or HIV services at this clinic today, about how much time did you spend waiting to be seen? This might include things like waiting at reception, or to see a health care provider. Do not include the time it took you to come to the clinic or the time it took you to get home. | Time to see clinicians<br>[ ][ ] : [ ][ ]<br>HOURS MINUTES<br>Time to get HIV test results<br>[ ][ ] : [ ][ ]<br>HOURS MINUTES<br>Don't know<br>Refuses to answer | <br><br><br><br><br><br>-1<br>-2 | |
| **D02** | Please tell me whether you agree or disagree with this statement: *The amount of time I have spent waiting during my clinic visit today was too long.* | Strongly agree<br>Agree<br>Neutral<br>Disagree<br>Don't know<br>Refused | 0<br>1<br>2<br>3<br>-1<br>-2 | |
| **D03** | How happy are you with the PrEP services that you have received at this clinic: very happy with the services, somewhat happy, or not at all happy? | Very happy<br>Somewhat happy<br>Not happy<br>Don't know<br>Refused | 0<br>1<br>2<br>-1<br>-2 | |

| No. | QUESTIONS & INSTRUCTIONS | RESPONSES | | SKIPS |
|---|---|---|---|---|
| **D04** | What are some of the things you like about this clinic? Select all that apply. | I do not like anything | 0 | |
| | | Staff are nice / helpful / welcoming | 1 | |
| | | Staff examine me well | 2 | |
| | | Staff explain things clearly | 3 | |
| | | Staff are knowledgeable about HIV /antiretroviral medicine | 4 | |
| | | Staff are discreet/ trustworthy | 5 | |
| | | Staff do not judge me | 6 | |
| | | Visits are quick / don't have to wait a long time | 7 | |
| | | Doctor / nurse spends sufficient time with me in discussing my care | 8 | |
| | | Clinic opening hours are convenient for patients | 9 | |
| | | Services are free / not expensive | 10 | |
| | | Facility is close by / convenient | 11 | |
| | | Facility is clean / nice | 12 | |
| | | Other_______________________ | 13 | |
| | | Don't know | -1 | |
| | | Refused | -2 | |

| No. | QUESTIONS & INSTRUCTIONS | RESPONSES | | SKIPS |
|---|---|---|---|---|
| **D05** | What are some of the things you do not like about this clinic? Select all that apply. | Staff are rude<br>Staff are unhelpful / not welcoming<br>Staff do not examine me well<br>Staff do not explain things clearly<br>Staff are not knowledgeable about HIV /<br>PrEP services<br>Staff are not discreet/ untrustworthy<br>Staff judge me<br>Visits are long / have to wait a long time<br>Doctor / nurse do not spend a lot of time<br>with me<br>Services are not free / expensive<br>Clinic opening hours are not convenient<br>Facility is far / inconvenient<br>Facility is dirty / bad<br><br>Other________________________<br>I like everything<br><br>Don't know<br>Refused | 1<br>2<br>3<br>4<br>5<br><br>6<br>7<br>8<br>9<br><br>10<br>11<br>12<br>13<br><br>14<br>0<br>-1<br>-2<br> | |
| **D06** | How comfortable are you with receiving PrEP services at this clinic? | Very comfortable<br>Somewhat comfortable<br>Not at all comfortable<br>Don't know<br>Refused | 0<br>1<br>2<br>-1<br>-2 | |
| **D07** | Would you agree that the health care workers in this facility are well trained and experienced in providing PrEP services? | Strongly agree<br>Agree<br>Neutral<br>Disagree<br>Don't know<br>Refused | 0<br>1<br>2<br>3<br>-1<br>-2 | |
| **D08** | Do you feel that your health care provider has answered all your questions and concerns about PrEP? | Yes<br>No<br>Don't know<br>Refused | 1<br>0<br>-1<br>-2 | |

| No. | QUESTIONS & INSTRUCTIONS | RESPONSES | | SKIPS |
|---|---|---|---|---|
| **D09** | Do you believe that sensitive information shared by patients in this clinic is kept confidential, and is not shared with or overheard by others who should not have access to this information? | Yes<br>No<br>Don't know<br>Refused | 1<br>0<br>-1<br>-2 | |
| **D10** | Would you agree that you feel safe and adequately free from stigmatizing attitudes in this clinic facility waiting area? | Strongly agree<br>Agree<br>Neutral<br>Disagree<br>Don't know<br>Refused | 0<br>1<br>2<br>3<br>-1<br>-2 | |
| **D11** | Have you experienced discrimination at this clinic facility during this visit? | Yes<br>No<br>Don't know<br>Refused | 1<br>0<br>-1<br>-2 | |
| **D12** | Would you recommend this clinic to friends seeking PrEP services? | Yes<br>No<br>Don't know<br>Refused | 1<br>0<br>-1<br>-2 | |

**Section F. Stigma and Discrimination Related to Key Populations (KP)**

**You will be asked about events that may have happened to you during this visit. For each item, please state how often this has happened to you because you are a sex worker, a man who has sex with men, transgender, or person who injects drugs. Please remember that there are no right or wrong answers and anything you say will be kept private and will not affect the care you receive for HIV. If any of these questions make you uncomfortable, just let me know and we can skip the question.**

| **F01**. During your visit, did the following events happen at the HIV testing clinic **because you are a sex worker, a man who has sex with men, transgender, or person who injects drugs?** | | **No** | **Yes** | | |
|---|---|---|---|---|---|
| **A** | I was verbally insulted/harassed | 1 | 2 | | |
| **B** | I was gossiped about by health facility workers | 1 | 2 | | |

| | | | | | |
|---|---|---|---|---|---|
| **C** | I received care I believe to be sub-standard | 1 | 2 | | |
| **D** | I was not treated with respect/dignity | 1 | 2 | | |
| **E** | Health care workers did not keep confidentiality | 1 | 2 | | |
| | **Section G. Patient feedback** | | | | |
| **G01.** | Lastly, we would be very interested to know any suggestions you have to improve care at this clinic. Please enter in the box any suggestions you have to improve the experience of receiving HIV care at this clinic. | | | | |

Thank you again for your time and help!
